# Progenitor cells of the rod-free area centralis originate in the anterior dorsal optic vesicle

**DOI:** 10.1186/1471-213X-9-57

**Published:** 2009-11-25

**Authors:** Sae Kyung Shin, Keely M Bumsted O'Brien

**Affiliations:** 1 Optometry and Vision Science, The University of Auckland, Auckland, NZ; 2 Visual Sciences, Research School of Biology, Australian National University, Canberra, ACT, AU

## Abstract

**Background:**

Nervous system development is dependent on early regional specification to create functionally distinct tissues within an initially undifferentiated zone. Within the retina, photoreceptors are topographically organized with rod free area centrales faithfully generated at the centre of gaze. How does the developing eye regulate this placement? Conventional wisdom indicates that the distal tip of the growing optic vesicle (OV) gives rise to the area centralis/fovea. Ectopic expression and ablation studies do not fully support this view, creating a controversy as to the origin of this region. In this study, the lineage of cells in the chicken OV was traced using DiI. The location of labelled cells was mapped onto the retina in relation to the rod-free zone at embryonic (E) 7 and E17.5. The ability to regenerate a rod free area after OV ablation was determined in conjunction with lineage tracing.

**Results:**

Anterior OV gave rise to cells in nasal retina and posterior OV became temporal retina. The OV distal tip gave rise to cells above the optic nerve head. A dorsal and anterior region of the OV correlated with cells in the developing rod free area centralis. Only ablations including the dorsal anterior region gave rise to a retina lacking a rod free zone. DiI application after ablation indicated that cells movements were greater along the anterior/posterior axis compared with the dorsal/ventral axis.

**Conclusion:**

Our data support the idea that the chicken rod free area centralis originates from cells located near, but not at the distal tip of the developing OV. Therefore, the hypothesis that the area centralis is derived from cells at the distal tip of the OV is not supported; rather, a region anterior and dorsal to the distal tip gives rise to the rod free region. When compared with other studies of retinal development, our results are supported on molecular, morphological and functional levels. Our data will lead to a better understanding of the mechanisms underlying the topographic organization of the retina, the origin of the rod free zone, and the general issue of compartmentalization of neural tissue before any indication of morphological differentiation.

## Background

A hallmark of nervous system development is the early fate determination of tissues followed by progressive morphological differentiation of initially featureless regions into cyto-architecturally and functionally distinct organs and systems. The onset of visual system formation begins with the establishment of tissue fated to become the retina, which occurs when the eye fields are inducted by a group of eye field transcription factors in the anterior neural plate [1,2]. Once the eye fields are established, rapid growth of the prosencephalon carries the eye fields laterally and anteriorly. The optic grooves (sulci) form when cells in the eye fields invaginate into the internal surface of the neural tube. As the anterior neural tube begins to close, the optic grooves deepen to become the optic vesicles (OV) [2]. Even though there are no obvious morphological specializations in the developing eye at the OV stage, it is at this point that the basic anterior/posterior and dorsal/ventral axes of the eye are established and positional coordinates forming a basic retinal map are assigned [3-6].

By the time the OV invaginates to form an optic cup, the tissues fated to be the neural retina and retina pigment epithelium (RPE) have been established. The retinal axes are further refined, which becomes critically important in the organization of many retinal cell types (reviewed in [2,7,8]). For instance, photoreceptor subtypes in many vertebrate species vary in their relative distribution across the retina (for review, see [9]). The chicken has a central retinal specialization, the rod-free area centralis that is faithfully generated at the centre of gaze along the horizontal retinal axis. Disruption of cells expressing spatially restricted genes, results in a loss or malformation of the photoreceptor topographic patterning [10]. Therefore it appears that the progenitor's location along the axes is important for determining a cell's fate.

How does the developing eye regulate the placement of the rod free area centralis? The perceived wisdom indicates that the first part of the growing OV to make physical contact with the overlying ectoderm, that is the distal tip, establishes the location of the area centralis. Once this region is established as the centre of the visual map, the basics divisions of the retina are formed [10,11]. However, there is no direct evidence showing that the rod free zone is generated from progenitor cells located at the distal tip of the OV.

In this paper, the hypothesis that the distal tip of the growing OV gives rise to the rod free area centralis was tested by lineage and ablation analysis. The data indicate that the chicken rod free area centralis originates from cells located near, but not at the distal tip of the developing OV. This anterior dorsal region, when ablated, produces a retina lacking the presumptive rod free zone with very little plasticity in the immediately surrounding OV regions observed. Taken together, these data do not support the hypothesis that the distal tip of the OV gives rise to the rod free area centralis; rather the data demonstrate that the origin of the rod free area centralis is in the anterior dorsal OV.

## Results

### Lineage Analysis

**Figure 1:**
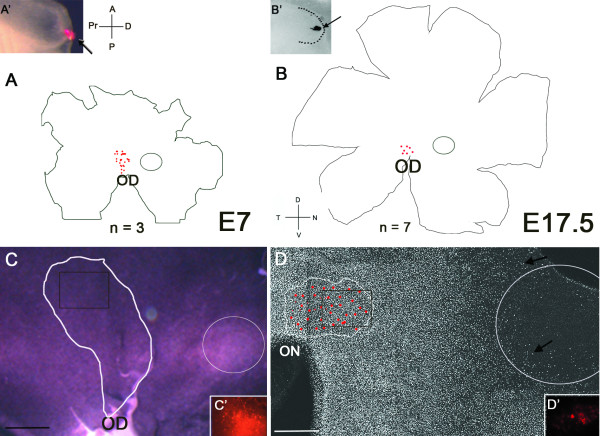
**Placement of DiI at the OV stage**. Placement of DiI on the optic vesicle (OV) distal tip leads to labelled cells above the optic disc (OD). (A') The insert shows the DiI placement at the OV distal tip. (A) Drawing of the characteristic labelling pattern in an E7 retina showing lineage traced cells above the OD (n = 3). (B') The insert shows a DiI placement (arrow) on the OV distal tip (outlined with dotted line). (B) A distal tip DiI placement at E17.5 leads to cells located above the OD (n = 7). (C) The region covered by DiI labelled cells is outlined in white on an image of an E7 retina processed for *in situ *hybridization for visinin. The black box corresponds to an insert showing an image of DiI labelled cells at E7.5. The visinin-free spot (circled) does not overlap with the lineage traced cells above the OD. (D) E17.5 retinal wholemount processed with a rod opsin antibody. Rod outer segments are visible as small white spots (arrows). The coverage of DiI lineage traced cells at E17.5 is outlined in white. The rod free zone (circled) does not overlap with the lineage traced cells above the OD. The black box corresponds to the area of DiI labelled cells shown in D'. Anterior (A), Posterior (P), Proximal (PR), and Distal (D) axes are indicated for the OV. Dorsal (D), Ventral (V), Nasal (N) and Temporal (T) are indicated for the retinal flatmounts. Scale bar in A and B = 1 mm; C = 0.5 mm and D = 50 μm.

The insertion of DiI crystals through the overlying ectoderm into the neural tube at the most distal region of the OV (Figure 1 A', B') was traced to a patch of retina above the optic disc (OD) containing DiI labelled cells at embryonic day (E) 7 and E17.5 (Figure 1A, B). With larger crystals (Figure 1A'), extensive bright patches of lineage traced cells constituted a central area with the density of labelled cells decreasing as the distance from the central bright patch increased (Figure 1C'). At E7, when the location of the visinin free spot (Figure 1C; open circle) was added to the map of the DiI labelled cells (Figure 1C, outlined area above optic disc), fate-mapped cells from the distal tip of the OV were always absent in the visinin free spot (n = 3; Figure 1C). At E17.5, the location of the rod free area centralis (open circle) was determined by labelling rod outer segments with an antibody to rod opsin (arrows; Figure 1D). In every case (n = 7), the DiI labelled cells (Figure 1D; red dots; 1D') were located above the optic nerve (outlined region with red dots) and were absent from the rod free zone (Figure 1D, open circle).

**Figure 2:**
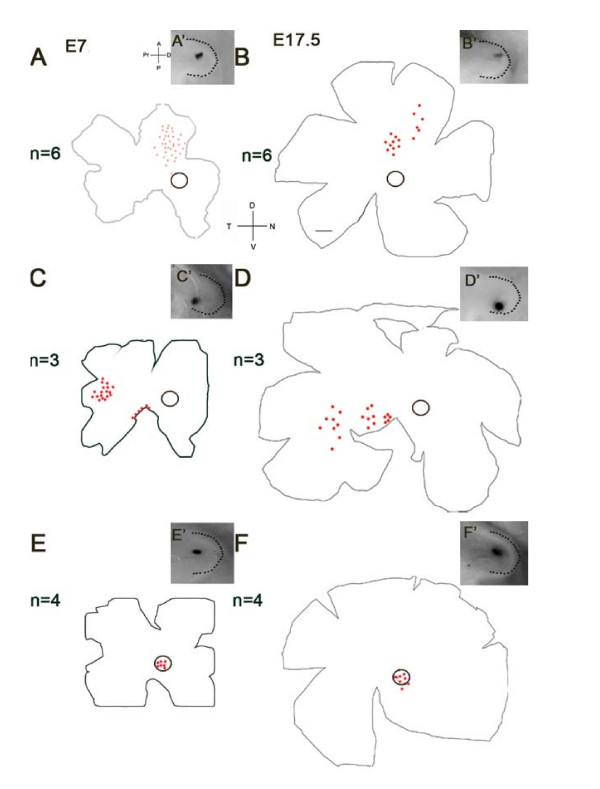
**Location of lineage traced cells after DiI placement**. (A', C', E') Images of optic vesicles (OV) (outlined with a dashed line) show the location of each DiI placement. (A-B) DiI placements in the anterior OV result in lineage traced cells being observed in the nasal retina at both E7 (n = 6; A) and E17.5 (n = 6; B). (C-D) There was no overlap with the visinin free spot (circle) at E7 or the rod free zone (circle) at E17.5. Posterior DiI placements lead to DiI positive lineage traced cells in the temporal retina at E7 (n = 3; C) and E17.5 (n = 3; D). There was no overlap with the visinin free spot (circle) at E7 or the rod free zone (circle) at E17.5. (E-F) Anterior dorsal placements of DiI lead to lineage traced cells in the visinin free spot at E7 (n = 4; E) and the rod free area centralis at E17.5 (n = 4; F). Anterior (A), Posterior (P), Proximal (PR), and Distal (D) are indicated for the OV. Dorsal (D), Ventral (V), Nasal (N) and Temporal (T) are indicated for the retinal flatmounts. Scale bar = 1 mm.

The anterior OV gave rise to more nasal retina at both E7 (n = 6; Figure 2A, A') and E17.5 (n = 6; Figure 2B, B'). The posterior OV gave rise to more temporal retina at E7 (n = 3; Figure 2C, C') and E17.5 (n = 3; Figure 2D, D'). For both the anterior and posterior DiI placements, the closer the placement to the distal tip, the more centrally located the cells, while DiI placements farther away from the distal tip mapped to more peripheral retina. More centrally placed DiI crystals led to a smaller area of lineage traced cells on the retina and more anterior or posterior DiI placements lead to a larger patch of lineage traced cells (Figure 2). Placement of DiI into the anterior dorsal OV resulted in labelled cells localized to the visinin free spot (circled) at E7 (n = 4; Figure 2E, E') and the rod free area centralis at E17.5 (n = 4; Figure 2F, F').

### Ablation Analysis

The survival rate of embryos after ablation was very poor when allowed to develop to E17.5 (approximately 15% survival). Although the eye grows significantly between E7 and E17.5, the visinin free spot at E7 was located at the same distance from the optic nerve head as the rod free area centralis at E17.5 (Figure 1 and 2). These data indicate that the location and complement of cells making up the rod free zone does not change between E7 and E17.5, thus allowing the location of the presumptive rod free zone to be analysed at E7. The survival rate of operated embryos was much higher when tissue was harvested at this earlier time (60% survival rate).

**Figure 3:**
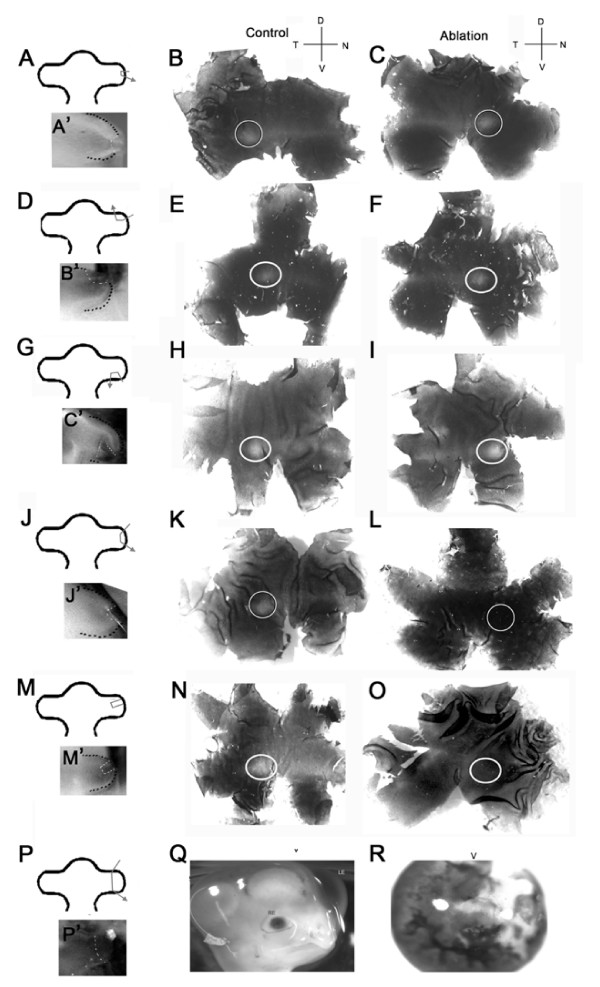
**Distal tip and regional ablations**. (A, D, G, J, M, P) Drawings and images of optic vesicles (OV) at time 0 (outlined with a back dashed line) indicate the location and extent of each ablation (white dashed line; A'-P'). The control non-ablated retina are shown on the left (B, E, H, K, N) and the ablated retina are shown on the right (C, F, I, L, O) after processing for *in situ *hybridization with a visinin probe. (A-C) A small distal tip ablation (n = 4) did not prevent the development of the visinin-free spot (circled) at E7. (D-F) Ablation of the anterior edge (n = 4) did not affect the development of the visinin free spot (circled). (G-I) Posterior OV edge ablations (n = 5) did not affect central patterning with the visinin-free spot (circled) observed in the control and ablated tissue. (J-L) A larger ablation (>20%) of the distal tip (n = 4) resulted in the loss of the visinin-free spot in the operated eye; compare circled regions in the control and ablated eyes. (M-O) Anterior dorsal ablations (n = 3) of less than 20% of the distal tip resulted in the loss of the visinin-free spot in the operated eye. (P-R) Removal of between 20-50% of the OV (n = 6) resulted in either small eyes (Q, right eye) compared to the non-operated eye (left eye) as shown, or pigmented blobs (R). Anterior (A), Posterior (P), Proximal (PR), and Distal (D) are indicated for the OVs. Dorsal (D), Ventral (V), Nasal (N) and Temporal (T) are indicated for the retinal flatmounts. Scale bar = 1 mm.

Small distal tip ablations did not disturb the development of the presumptive rod free zone, as indicated by the presence of a visinin free spot in the control and operated eye (Figure 3A-C). When a small ablation covering less than one third of the anterior (n = 4; Figure 3 D-F) or posterior (n = 5; Figure 3G-I) OV was removed, there was no loss of the visinin free spot at E7. The visinin free spot was lost only when a larger portion of the distal tip (still less than 20% of the total OV) including the anterior dorsal region fate mapped to become the cells in the rod free area centralis (n = 4; Figure 3 J-L) or a significant amount of the region anterior and dorsal to the distal tip of the OV was removed (n = 3; Figure 3 M-O). When more than 20% of the OV was removed (Figure 3P), the size of the eye and the survival rate significantly decreased. The example provided in Figure 3 Q-R shows the results when 50% of the OV is removed, resulting in a small pigmented blob/small eye (n = 6).

### Cell movements along the anterior posterior and proximal distal axes

**Figure 4:**
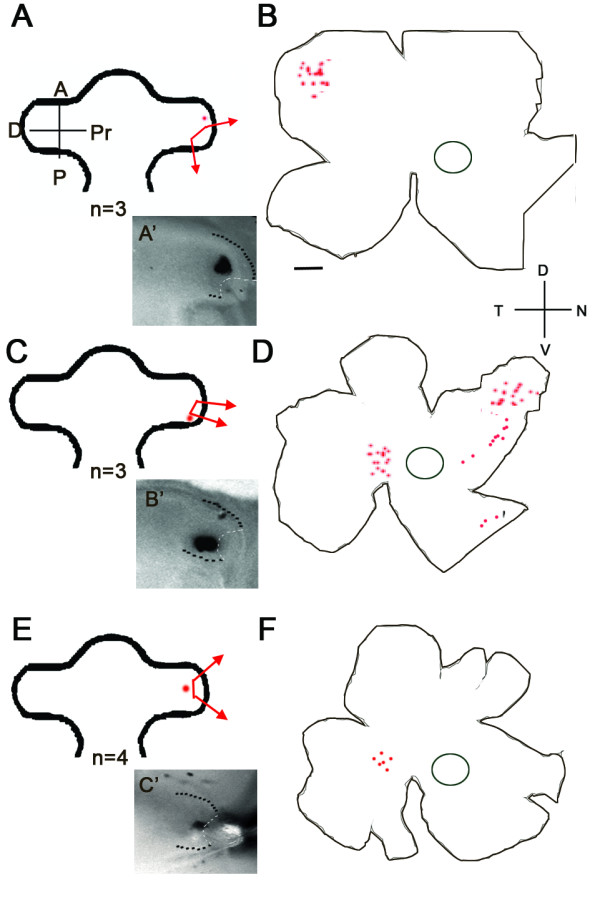
**Lineage tracing after OV ablation**. (A, C, E) The location of the ablation and subsequent DiI placement is shown in an image of an OV (outlined in black) with an ablation (outlined in white; A', B', C'). The anterior (a), posterior (p) and the distal (d) and proximal (pr) axes are indicated. (A-B) A posterior ablation of the OV with the DiI placed on the dorsal edge near the distal tip led to a small peripheral dorsal region containing DiI labelled cells at E7. (C-D) Placement of the DiI more proximally toward the posterior edge of the ablation led to a large region of lineage traced cells spread over to distinct areas. (E-F) DiI placed on the dorsal distal edge of a distal tip ablation lead to a small region of retina containing lineage traced cells. Anterior (A), Posterior (P), Proximal (PR), and Distal (D) are indicated for the OV. Dorsal (D), Ventral (V), Nasal (N) and Temporal (T) are indicated for the retinal flatmounts. Scale bar = 1 mm.

**Figure 5:**
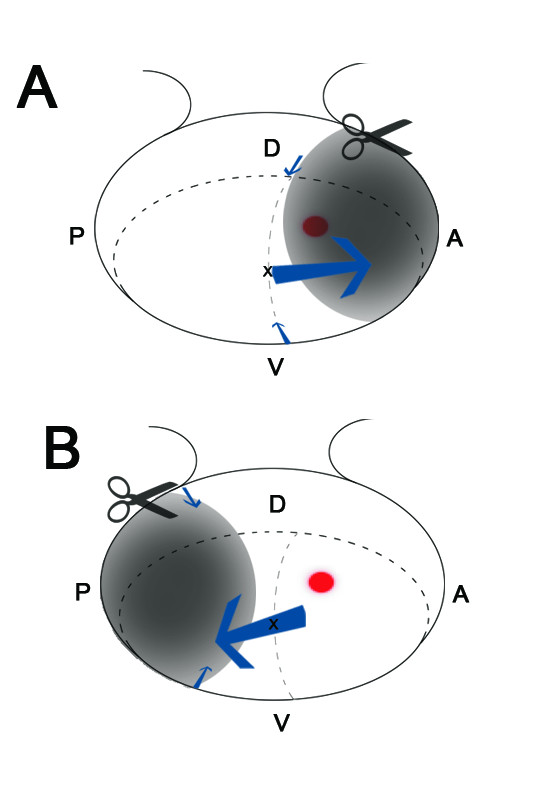
**Summary of Ablation and Cell Movements**. (A) A large anterior ablation (scissors and shaded area) that includes the area giving rise to the rod free zone (red dot) is healed by the movement of cells along the anterior/posterior axis (large blue arrow), with little contribution from the dorsal/ventral regions (small blue arrow). In this experiment, the rod free zone is lost and the cells that would normally reside more centrally are located in the far nasal periphery, which corresponds to the anterior optic vesicle. (B) A large posterior ablation (scissors and shaded area), which does not include the rod free zone (red dot) is healed by the incorporation of cells from central and anterior regions along the anterior posterior axis (large blue arrow), with little contribution from the dorsal/ventral axis (small blue arrows). In this experiment the rod free zone remains and the retina has acquired an anterior identity.

DiI crystals were placed on the anterior edge of the wound following a posterior ablation (n = 3; Figure 4A), the posterior wound edge of a smaller posterior ablation nearer to the distal tip (n = 3; Figure 4C), and the proximal wound edge of a distal tip ablation (n = 4; Figure 4E). With the larger posterior ablation, DiI labelled cells originating along the wound edge in the anterior OV were located in the peripheral temporal retina at E7 (Figure 4B). In intact OVs, DiI labelled cells from this region were located more centrally in nasal retina at E7 (Figure 2), which indicated that the cells along the wound edge expanded posteriorly to heal the ablation. DiI placed on the posterior wound edge of a small posterior OV ablation were found in the temporal retina, as expected, as well as in a large area of the dorsal peripheral nasal retina (Figure 4D). In a non-operated OV, DiI placed in this posterior location were found in cells located in mid peripheral temporal retina (Figure 2), again indicating that there were major cell movements from the wound edge along the anterior posterior axis. The DiI labelled progenitor cells next to the proximal wound edge of the distal tip ablation were observed in the temporal mid-peripheral retina along the horizontal meridian (Figure 4F), similar to what was observed with fate mapping without ablation (Figure 2). These data indicate that there was a great deal of cell movement along the anterior/posterior axis during the wound healing process and there was minimal movement of cells along the distal/proximal axis (Figure 5).

## Discussion

This study shows that the progenitor cells which give rise to the chicken rod-free area centralis originate from a region in the anterior dorsal OV, close to, but not at, the distal tip. The cells located at the distal tip of the OV are fated to differentiate into cells located in the central retina above the optic nerve head. Lineage analysis indicates that the visinin free spot observed at E7 is localized to the same retinal region as the rod free area centralis at E17.5. When a small amount of distal tip is ablated (less than 20%), the presumptive rod free zone is maintained, but if more than 20% is ablated, the rod free zone is lost. Ablations removing more than 50% of the OV give rise to pigmented blobs or small eyes. Only when an ablation removes a significant amount of the dorsal anterior region is the rod free zone lost (Figure 5A). After ablation, labelled cells at the wound edge showed significant migration along the anterior/posterior axis, but minimal migration along the dorsal/ventral axis (Figure 5). Therefore, a general characteristic of the developing nervous system, the progressive compartmentalization of the initially morphologically undifferentiated tissues, is clearly observed in the growing eye anlage and this pattern is critical for regulating regional plasticity and topographic organization.

### The OV distal tip gives rise to the region above the optic nerve

Our data suggests that the distal tip of the OV is important for identifying the location of the developing optic nerve; rather than the formation of the rod free zone. During the transition from OV to optic cup, once the distal tip of the OV reaches the overlying ectoderm, the expanding OV begins to invaginate to form a cup-shaped structure, fold along its center line, and to acquire regionally specific characteristics [2,12-14]. The ventral optic cup then grows around the optic stalk to form the optic fissure allowing the entrance of mesenchymal cells to form the hyaloid artery [15-18]. As the optic fissure seals, the first retinal ganglion cells exit from the eye, thus forming the optic nerve. Disruptions to this process result in a failure of the optic cup to form or a failure of the optic fissure to close leading to the formation of a coloboma [19-22]. The fate mapping of cells from the distal tip of the OV to the region above the optic nerve indicates that this region may be important for the formation of the optic stalk and optic fissure, rather than the formation of the rod free area centralis.

### The Rod Free Area Centralis Originates in the Anterior Dorsal OV

The assumption that the distal tip of the OV gives rise to the rod free area centralis stems from a lineage analysis paper by Dutting and Thanos who demonstrated that the distal tip of the OV gives rise to a large proportion of the central retina, above or surrounding the optic nerve head [4]. This hypothesis is further supported by a series of OV ablations followed by analysis of the rod free area centralis performed by Schulte et al. [10]; however, lineage tracing was not included after these ablations. The overall pattern of regional cell lineage observed in our experiments agrees with Dutting and Thanos (see injection sites 4, 5 and 13), with our data providing a more detailed picture of the lineage analysis of the central retina. With this more detailed map taken into account, the ablations reported by Schulte and colleagues demonstrate that only the loss of a more anterior dorsal region of the OV leads to the loss of the rod free zone [10]. There is little plasticity in the developing optic cup to regenerate the rod free area centralis once ablated.

The localization of the rod free progenitor zone to an anterior dorsal region is further supported by changes in regional gene expression in the retina after manipulation of the anterior/posterior and dorsal/ventral axes by ablation or ectopic expression. Anterior OV ablation results in the posterior vesicle remnant reconstituting a normal-sized retina with posterior characteristics, which lacks the rod free area centralis (Figure 5A) [10]. Similar to our results (Figure 5B), Schulte and colleagues showed that gene expression changes or ablations which emphasize the anterior OV do not influence the generation of the rod free zone [10]. This is likely due to the fact that the progenitors of the rod free area are located in the anterior optic cup. Consistent with our data, previous studies have shown that loss of the dorsal OV, either through ablation or ectopic expression of the ventrally expressed genes leads to an expansion of ventral characteristics in cell topography- including the loss of the rod free zone [10,23]. Disruption of the ventral retina by the ectopic expression of the dorsal specific genes does not affect the generation of a rod free zone; rather the extent of the rod pattern is perturbed [10].

### Limits of OV/Eye Regeneration after ablation

Previous studies using partial ablation of either the anterior or posterior OV indicate a range of resulting eye morphologies, ranging from normal sized eye, to microphthalmic eyes to pigmented blobs [3,10,24,25]. Hirashima and colleagues showed that ablation of small regions of anterior OV did not interfere with the development of a normal eye [25], which is similar to our observations. We observed that only the removal of a small anterior dorsal region resulted in the lack of a rod free zone; consistent with the finding that not all regions of the developing OV and cup are capable of giving rise to the rod free zone [25]. In our ablation experiments where a small eye was formed, it is likely that the majority of tissue removed was dorsal OV, leaving the ventral OV, which has the capacity to develop into an entire eye structure.

### Major movements of cells along the anterior posterior axis

Healing of the OV after ablation requires cell movements and increased proliferation to grow the two edges of the wound together. It is likely that there is a difference in proliferation and a limit to plasticity at the edges of the wound. Dutting and Thanos suggested that the cells located in the peripheral anterior or posterior OV give rise to the larger retinal area in the E6 retina, suggesting that retinal progenitor cells located in most anterior and posterior OVs undergo increased proliferation [4]. The increased wound healing potential of cells along the anterior/posterior axis (large arrows; Figure 5) and the decreased proliferation in the central regions along the dorsal ventral axis (small arrows; Figure 5) limits the ability to regenerate the rod free zone. In addition, Peters and Cepko argue that there is an intrinsic difference between the dorsal/ventral and anterior/posterior axes with retinal clones remaining in their dorsal or ventral domains [16]. Our observation that the major movements of cells following ablation during the healing were along the anterior/posterior axis (Figure 5) indicates that a clone has a greater probability of generating daughter cells along the anterior/posterior axis compared with the dorsal/ventral axis indicating an increased potential for plasticity. Therefore, the relatively smaller area of the central retina arising from a more dorsal and anterior location leads to decreased proliferation potential and a restriction of plasticity of the rod free zone (Figure 5).

### Visinin free spot is a good indicator of the presumptive rod free zone

During retinal development, there is a correlation between the location of the visinin free spot at E7 or the rod free area centralis at E17.5 and the optic nerve. This consistent distance has been observed in the human retina, with the fovea located 4.9 mm from the optic nerve head with little variability between eyes and individuals [26]. In addition to the loss of visinin in the presumptive rod free zone, a number of other genes are specifically localized to this region [27-30]. Therefore, the highly conserved distance between the OD and the visinin free spot or the rod free zone, as well as the exclusion or inclusion of regionally specific genes (reviewed in [8]) can provide clues to identifying that part of the developing retina and indicates that the region of the retina forming the rod free area dorsalis has a distinct molecular signature that differs from the surrounding retina.

## Conclusion

Taken together, our data indicates that the hypothesis that the area centralis is derived from cells at the distal tip of the OV is not supported; rather, a region anterior and dorsal to the distal tip gives rise to the rod free zone. The location of the rod free zone is specified prior to E7 and its location remains constant in relation to the OD throughout the rest of development. When compared with other studies of retinal development, our results are supported on molecular, morphological and functional levels. Our data will lead to a better understanding of the mechanisms underlying the topographic compartmentalization of the retina and the origin of the rod free zone as well as lay the foundation for additional large scale investigations of the molecules controlling the formation of the rod free zone. These results are applicable to a wider audience in that they show the early compartmentalization of neural tissue before any indication of morphological differentiation. The conserved mechanisms that function to generate these distinct regions should be studied more closely as the results will further the development of an accessible and highly informative model for neural differentiation.

## Methods

### Cell lineage analysis and surgical manipulation of embryos

All experiments were performed and tissue collected with ethical approval from the Australian National University and the University of Auckland Animal Ethics committees. Fertilized chicken (*Gallus gallus*) eggs obtained from a local breeder were incubated at 37.8°C in a humidified incubator for 36-48 hours. A window was made in the top of the egg above the developing embryo. The vitelline membrane covering the embryo was removed and India ink diluted in sterile 0.1 M Phosphate buffered saline (PBS), pH 7.4 was injected beneath the embryos to visualize the embryonic structures and to allow the embryos to be staged according to Hamburger [31]. Embryo lineage analysis was begun with embryos at Hamilton Hamburger (HH) stage 10-11 (9-13 somites) [31]. The OV was supported by placing a metal probe underneath. Lipophilic DiI crystals were implanted manually or crystals dissolved in peanut oil were injected using a microcapillary attached to an Eppendorf cell tram (Eppendorf South Pacific, North Ryde, Australia) into the developing OV at the distal tip, and in various dorsal anterior and dorsal posterior regions.

Partial removal (ablation) of the OV was performed at HH stage 10-11. Using sharpened tungsten needles, varying amounts of the right OV were ablated. The left OV served as a control. For cell lineage analysis following ablation, a DiI crystal was implanted on the cut edge using sharp tungsten needle. After ablations, and ablations with DiI placements, 250 μL 0.1 M phosphate buffered saline (PBS) was added to the top of each embryo, the embryos were photographed with a Leica camera (Leica GmbH, Germany) attached to a Leica dissecting microscope, the windowed egg was sealed with tape, and all eggs returned to the incubator until either E7 or E17.5. The earlier age, E7, was chosen because this is similar to the age used for previous chicken OV fate mapping studies, therefore allowing a comparison to be made with well established data [4]. The older age of E17.5 was chosen because the location of the rod free zone was determined by labelling with a rod opsin antibody.

### Tissue Collection and Preparation

At E7 (HH stage 31) or E17.5 (HH stage 44), the embryos were removed from the shell, quickly decapitated and the eyes enucleated. The cornea, lens and vitreous were removed. The retina was then dissected away from the sclera and RPE. The retina was fixed overnight in 4% paraformaldehyde, and then washed twice in 1× PBS for 5 minutes. The retina was flattened by making several incisions along the retinal margins and then whole-mounted on a glass slide and photographed on a Leica (Leica, Germany) dissecting microscope. Because the DiI labelling was not retained in the cells after *in situ *hybridization or immunohistochemical processing due to the methanol (MeOH) step, the DiI containing fluorescing cells were located and photographed using a Leica compound microscope (Heidelberg, Germany). The location of the DiI labelled cells was determined by measuring and recording the distance from known retinal landmarks (e.g. optic nerve, retinal edge).

The retina was then transferred back into PBS and then dehydrated in an ascending series of PBS containing 0.1% Tween-20 (PBT) and MeOH. Retinas were stored in 100% methanol at -20°C until use.

### *In situ *Hybridization

To begin the *in situ *hybridization protocol, the E7 retinas were rehydrated through a descending PBT/MeOH series. Once in PBT, any remaining pigment epithelium was bleached [32,33]. The retinas were then sealed between nylon or silk meshes. For the initial experiments, nylon mesh was used; however the nylon mesh pattern was imprinted on the tissue. Silk does not leave an imprint and was used in subsequent experiments.

*In situ *hybridization was carried out at E7 using digoxigenin-labeled visinin riboprobes, as described previously [34,35]. Visinin, a small calcium binding protein expressed in chicken photoreceptors, is excluded from the developing area centralis at E7 [34]. The visinin-free spot becomes visible at E7 (4-6 scleral papillae) and correlates with the location of the presumptive rod free zone. Hybridisation of labelled probe was visualised using alkaline phosphatase conjugated anti-DIG antibody and the colour reaction was developed with NBT/BCIP (Roche, Germany). Retinas were mounted photoreceptor layer up on glass slides and mounted in 80% glycerol/PBS. Bright field images were taken with Leica microscope, and subsequently imported into Adobe Photoshop CS2 and adjusted for color balance and sharpness.

### Wholemount Immunohistochemistry

Wholemount immunocytochemistry on the E17.5 retinas was performed as previously described [36]. Briefly, retinas, stored in MeOH were rehydrated through a descending PBT/MeOH series. Once in PBT, any remaining pigment epithelium was bleached [32,33]. After three 30 minute washes in PBS, the primary monoclonal anti-rod opsin antibody (RETP1; 1/50) diluted in 1xPBS, 3% Bovine Serum Albumin and 0.1% Triton was added to each retina. The retinas were incubated in the primary antibody for 4 days, followed by three 30 minute washes in PBS. The secondary antibody Alexa 488 goat anti mouse (1:1000) was added and the tissue incubated for 24 hours at 4°C. The retinas were then washed three times in PBS for five minutes each wash. Following the last PBS wash, retinas were mounted photoreceptor layer up on glass slides in 80% glycerol/PBS. Bright field images were taken with a Zeiss LSM 5 PASCAL confocal microscope system and PASCAL version 4.0 software (Jena, Germany), imported into Adobe Photoshop CS2 and adjusted for color balance and sharpness.

### Wholemount Mapping

Slight changes in the shape of the retina were observed after the tissue processing for *in situ *or immunohistochemistry; however the overall shape of the wholemount remained similar to that recorded when the DiI labelled cells were mapped. A schematic representation of the regional labelling of the DiI cells was mapped onto a sketch of the flat-mounted retina. The location of the visinin free spot or the rod free zone was recorded in relation to the optic nerve, and then this information was included in the map containing the location of the DiI labelled cells. Any slight changes in shape or size of the flatmount that were observed when comparing the map of the DiI labelling and post *in situ *or immunohistochemistry were noted.

## Authors' contributions

SKS assisted in the design of the project and carried out the lineage analysis and ablation studies, followed by the *in situ *hybridization experiments, and drafted the manuscript. KMBO supervised the design and implementation of the project, assisted with the lineage and ablation analysis, performed the immunohistochemistry experiments, analysed the results, critically revised the manuscript, and gave final approval for this version to be published. All authors read and approved the final manuscript.
